# Optimal power flow using hybrid firefly and particle swarm optimization algorithm

**DOI:** 10.1371/journal.pone.0235668

**Published:** 2020-08-10

**Authors:** Abdullah Khan, Hashim Hizam, Noor Izzri bin Abdul Wahab, Mohammad Lutfi Othman

**Affiliations:** 1 Department of Electrical and Electronic Engineering, Universiti Putra Malaysia, Selangor, Malaysia; 2 Advanced Lightning, Power and Energy Research (ALPER), Faculty of Engineering, Universiti Putra Malaysia, Selangor, Malaysia; Sunway University, MALAYSIA

## Abstract

In this paper, a novel, effective meta-heuristic, population-based Hybrid Firefly Particle Swarm Optimization (HFPSO) algorithm is applied to solve different non-linear and convex optimal power flow (OPF) problems. The HFPSO algorithm is a hybridization of the Firefly Optimization (FFO) and the Particle Swarm Optimization (PSO) technique, to enhance the exploration, exploitation strategies, and to speed up the convergence rate. In this work, five objective functions of OPF problems are studied to prove the strength of the proposed method: total generation cost minimization, voltage profile improvement, voltage stability enhancement, the transmission lines active power loss reductions, and the transmission lines reactive power loss reductions. The particular fitness function is chosen as a single objective based on control parameters. The proposed HFPSO technique is coded using MATLAB software and its effectiveness is tested on the standard IEEE 30-bus test system. The obtained results of the proposed algorithm are compared to simulated results of the original Particle Swarm Optimization (PSO) method and the present state-of-the-art optimization techniques. The comparison of optimum solutions reveals that the recommended method can generate optimum, feasible, global solutions with fast convergence and can also deal with the challenges and complexities of various OPF problems.

## 1 Introduction

Electric services companies are repeatedly working for generation scheduling and reasonable operational state to optimize the generating cost based on effective security limits and power transfer confinements. The optimal power flow (OPF) is an essential and complex optimization technique in electrical power system operations to adjust and optimize the control settings with various constraints sit [1] [2] [3] [4]. The earliest, various conventional optimization techniques have been used to solve the OPF problems. Main objective of the OPF problem to obtain the optimize scheduling of particular control variables based on limitation of system constraints sit [5] [6] [7]. These constraints consists of equality and inequality constraints. Equality constrains includes power flow or balance equations, whereas the inequality constrains sphere the dependent and decision variables within its limits.

Newly, single and multiple objective OPF techniques have been developed to obtain optimized solutions based on technical and economic interests. Many developers applied conventional and recent optimization techniques to deal with the OPF problems. there conventional optimization algorithms are: non-linear programming sit [8] [9], decomposition algorithms sit [10], the Newton algorithm sit [11], and quadratic programming sit [12] to solve the OPF problems. Linearization of constraints and specific objective function are main drawback that effects the final solution. Many limitations of the conventional OPF are mentioned in sit [13]. Complete review of the mentioned classical optimization methods is presented in sit [14]. New techniques with critical aspects and new advance are suggested for OPF problems sit [15].

Recently, Notable progress in the field of digital computation, artificial intelligence algorithms combined with nature-inspired, meta-heuristic based optimization methods are used to help electrical system based on economic concern. Numerous heuristic-based optimization algorithms have been proposed and applied to handle OPF problems, such as genetic algorithm (GA) sit [16] [17] [18]. In addition, many methods were developed to improve global performance and convergence of GA method, such as adaptive genetic algorithms with adjusting population size (AGA-POP) sit [19] and enhanced GA sit [19].

Newly developed search-based optimization algorithms are applied for OPF problems, like particle swarm optimization (PSO) method sit [2], differential evolutionary technique sit [20] [21]), improved colliding bodies optimization method sit [22], improved PSO algorithm sit [23], biogeography-based optimization technique sit [24], imperialist competitive method sit [25], grey wolf optimizer sit [26], hybrid algorithm of PSO and GSA algorithm sit [27], differential search technique sit [28], gravitational search method (GSM) sit [29] [30] [31], multi-phase search optimization technique sit [32] [33], fuzzy-based hybrid PSO algorithm sit [34], chaotic self-adaptive differential harmony search method sit [35], black-hole-based optimization technique sit [36], harmony search technique sit [37], artificial bee colony method (4), Jaya optimization technique sit [38], teaching-learning-optimization algorithm sit [39], biogeography-based optimization (BBO) sit [40], differential evolution (DE) sit [41], artificial bee colony (ABC) algorithm sit [42], distributed algorithm (DA) sit [43], and the Firefly algorithm (FA) sit [44]. An analysis of a non-deterministic algorithm, which is applied to solve OPF, is mentioned in sit [45]. Unfortunately, some of these methods are not effective for global optimization of various OPF problems, through a simultaneous calculation of various points in the search space. Such population-based, meta-heuristics algorithms are more efficient, compared to trajectory techniques, to find local optima. On the other hand, the trajectory techniques are good at describing global optima. Hence, hybridization of these meta-heuristic methods can use the benefits of both methods and can deal with more complex and challenging problems because of their robustness and flexibility sit [46].

The key goals of the hybrid meta-heuristic particle swarm optimization algorithm modifications are to create equilibrium between exploration and exploitation and to escape from premature results. Additionally, hybridization can improve the PSO’s capability and eliminate its weakness sit [47]. The main advantages of the PSO method are fast convergence, less calculating resource necessities, and easy implementation. But when populations are near to each other sit [48], this method suffers from being confined in local optima and by slow convergence. The Firefly optimization method is also a nature-inspired optimization method that copies the behavior of fireflies. It has some specific benefits over the PSO algorithm sit [49]. One of the benefits is that it does not have local or global best variables, so this helps it from being caught up in local optima. The method also doesn’t have a velocity vector, so it can prevent the problems that are created by the variations in velocity sit [50].

One of the recently developed hybrid meta-heuristic, population-based optimization methods entitled Hybrid Firefly Particle Swarm Optimization (HFPSO), developed by Aydilek İB sit [51]. Some real engineering problems have been tested on the HFPSO algorithm and the results have been compared to present-day state-of-the-art optimization algorithms. The overall results confirmed that the HFPSO method has the power to provide promising results that were not explored before sit [51]. The use of the HFPSO method to solve the OPF problems had not been studied. Hence, applying a robust optimization method can efficiently overcome the OPF problems.

This article proposed using the Hybrid Firefly Particle Swarm Optimization (HFPSO) method first, to contribute and solve various OPF problems in the power-engineering field. An expanded set of variables is used in the suggested OPF formulations. The set consists of actual power and voltages of generating units, transformer turn ratios, and reactive power of Shunt VAR compensators.

Five single-objective functions are considered in this article to show the efficiency of the proposed method considering optimum results of OPF problems: total generation cost minimization, voltage profile improvement, voltage stability enhancement, active and reactive power transmission loss reduction.

The improved performance is shown by comparing the results of the proposed HFPSO algorithm with the state-of-the-art algorithms chosen from the current literature for OPF problems. The proposed algorithm is also compared with its mother PSO algorithm, from which it is derived. The same single-objective OPF problems were used in the above-mentioned algorithms for the comparison. The standard IEEE 30-bus test scheme is applied to observe, authenticate, and show the effectiveness of the HFPSO algorithm.

The key contributions of this paper are as follows:

1. This work proposes an already developed HFPSO algorithm to tackle the OPF problems.
2. The algorithm is applied to five single-objective functions of OPF problems.
3. Various objective functions of OPF problems are considered, such as total fuel cost minimization, voltage profile improvement, voltage stability enhancement, active and reactive power losses reduction.
4. Results of the proposed algorithm are compared with simulated results of PSO and current literature work. So, these compressions prove supremacy of the algorithm in terms of convergence ratio and optimal results based on OPF problems.
5. Statistical analysis showed that HFPSO algorithm is a robust and reliable optimization method to solve OPF problems.

The rest of this paper is organized as follows: Mathematical formulation of OPF issues is given in Part 2. Part 3, 4, and 5 briefly explain PSO, FOA, and HFPSO algorithms, respectively. Part 6 summarizes application of the proposed HFPSO algorithm to the OPF problems. Results, comparison, and discussion are explained in Part 7. Conclusions about the application of the HFPSO algorithm are mentioned in Part 8.

## 2 Problem formulation

Five cases, with five objectives, are considered in this study to verify the efficiency of the proposed HFPSO technique regarding optimum results of OPF problems. The objectives are total fuel cost minimization of the power network, voltage profile improvement, reduction of the active power losses of transmission lines, reduction of reactive power losses of transmission lines and voltage stability enhancement. Fuel cost *f*_1_ of a particular electrical power system is characterized by subsequent functions sit [39]:

$$\begin{array}{c} f_{1}=\sum_{i=1}^{N_{G}}f_{i} \end{array}$$ (1)

Where *N*_*G*_ represents the number of power generating units and the fuel cost of the *i* − *th* power-generating unit is denoted by *f*_*i*_, the quadratic function *f*_*i*_ is formulated as follows:

$$f_{i}=b_{i}\left(P_{Gi}\right)+c_{i}\left(P_{Gi}\right)+c_{i}{\left(P_{Gi}\right)}^{2}\left(\$/hr\right)$$ (2)

Where *a*_*i*_,*b*_*i*_, and *c*_*i*_ are coefficients of fuel price of the *i* − *th* power generating unit and *P*_*Gi*_ output active power of the *i* − *th* generator unit. The bus voltage is one of the key indicators for security and service quality indices sit [41]. To avoid the infeasibility, a double objective function, such as improvement of voltage profile and reduced fuel cost are considered as a single-objective function in the OPF issue. The objective task *f*_2_ is stated as sit [52]:

$$\begin{array}{c} f_{2}=\sum_{i=1}^{N_{G}}f_{i}+c\sum_{i=NL}\left|V_{i}-1.0\right| \end{array}$$ (3)

Where *c* is used as a weight factor for the stability between the objectives to avoid the dominance of one function over the other.

Due to economic reasons, a transmission network of a power system is mandatory to function near its security boundaries. The stability of a power system is one of the very important domains, to limit the bus voltage at every single point below standard working conditions during the load surge. The disturbance leads to changes in the system’s configuration. Consequently, an unavoidable voltage collapse accrues sit [39]. Voltage balance of a specific power network can be indicated by using *L* − *index*, that is *L*_*max*_ sit [53].

$$L_{max}=max\left[L_{a}\right],a=1,2,3,\ldots \ldots ,N_{L}$$ (4)

Where *L*_*a*_ denotes the *L*_*max*_ of *a* − *th* demand bus and *N*_*L*_ is the integer of *PQ* (demand) buses, the objective function *f*_3_ of the case is represented as follows sit [39]:

$$\begin{array}{c} f_{3}=\sum_{i=1}^{N_{G}}f_{i}+w\left|L_{max}\right| \end{array}$$ (5)

Active power line transmission losses are a very important factor to optimize in a power network. The objective work *f*_4_ is denoted by the power balance equation in this case sit [36].

$$\begin{array}{c} f_{4}=\sum_{i=1}^{NLB}P_{i}=\sum_{i=1}^{NLB}P_{Gi}-\sum_{i=1}^{NLB}P_{Di} \end{array}$$ (6)

Where *P*_*i*_ is the active transmission line power loss, *P*_*Gi*_ is the active power of a generating unit and *P*_*Di*_ the active power of the request (demand) of the *i* − *th* load line.

The availability of reactive power is an important factor in consideration of the voltage balance margin of a static power network, to reinforce the conduction of active power from the generator to the load. Thus, the optimization of reactive power losses can be stated by the following equation sit [36]:

$$\begin{array}{c} f_{5}=\sum_{i=1}^{NLB}Q_{i}=\sum_{i=1}^{NLB}Q_{Gi}-\sum_{i=1}^{NLB}Q_{Di} \end{array}$$ (7)

Where *Q*_*i*_ is the reactive transmission line power loss, *Q*_*Gi*_ is the reactive power of source and *Q*_*Di*_ the reactive power of demand of the *i* − *th* load line.

As aforementioned, OPF provides optimal tuning of the control variables of demand or load to minimize a preset objective task, such as the total cost of a power system or active and reactive transmission line power losses. Most of the OPF detail may be characterized by the standard method sit [14]:

$$\begin{array}{c} \text{Minimize}\begin{array}{c} x=(g,h) \end{array} \end{array}$$ (8)

$$\begin{array}{c} \text{Subjected}\;\text{to}\begin{array}{c} u(g,h)=0 \end{array} \end{array}$$ (9)

$$\begin{array}{c} \text{And}\begin{array}{cc} z(g,h) & x2264;0 \end{array} \end{array}$$ (10)

Where *h* denotes the vector of control variables and *g* denotes the vector of stated variables, *x*(*g*, *h*) states the system’s objective function. *u*(*g*, *h*) and *z*(*g*, *h*) indicates the sets of equality and inequality constraints. Also, the dependent *h* and the independent *g* variables of the OPF problems are detailed in (11) and (12) separately. The control variable *h* can be stated as sit [54] [39]:

$$h=[P_{G2},\ldots \ldots P_{NG_{GG}},T_{1},\ldots \ldots T_{N_{TT}},V_{G}1,\ldots \ldots V_{N_{GG}},Q_{C}1,\ldots \ldots Q_{N_{CC}}{]}^{T}$$ (11)

Where *P*_*G*_ stands for the active power generation at the *PV* (generator) buses apart from the swing bus, *T* represents the tapping ratio of the transformer. *V*_*G*_ refers to the voltage value at generator buses, *Q*_*C*_ denotes the reactive power injection by shunt capacitor respectively. Moreover, *N*_*GG*_, *N*_*TT*_, and *N*_*CC*_ represent the number of generator units, regulating transformer units and shunt capacitor units. The state of an electrical network can be represented by OPF formulations sit [10]. The most common, dependent variables for OPF issue are formulated along these lines sit [54] [39]:

$$g=[P_{Slack},V_{L1},\ldots \ldots V_{LN_{L}},Q_{G1},\ldots \ldots Q_{GN_{G}},S_{ln1},\ldots \ldots S_{ln_{N}}{]}^{T}$$ (12)

Where *P*_*Slack*_ shows the active power generation of the swing bus, *V*_*L*1_ denotes the voltage value at *PQ* or load buses. *Q*_*G*_ symbolizes the reactive power of generators, and *S*_*ln*_ denotes the line flow and line loading, respectively. Furthermore, *N*_*L*_ and *N* are the integers of *PQ* buses and power lines, correspondingly.

OPF constraints can be categorized into two types: 1) equality and 2) inequality constraints. The equality constraints of the OPF show the physical condition of a power network sit [54] [39]:

$$\begin{array}{c} P_{Gi}-P_{Di}=V_{i}\sum_{i=1}^{N}V_{j}\left(G_{ij}cos_{ij}\right)+\left(B_{ij}sin_{ij}\right) \end{array}$$ (13)

$$\begin{array}{c} Q_{Gi}-Q_{Di}=V_{i}\sum_{i=1}^{N}V_{j}\left(G_{ij}sin_{ij}\right)+\left(B_{ij}cos_{ij}\right) \end{array}$$ (14)

Where *P*_*Gi*_ and *Q*_*Gi*_ represent the real and imaginary parts of the creation of a power network, *P*_*Di*_ and *Q*_*Di*_ are the real and imaginary parts of the network demands on the *i* − *th* bus. Moreover, *B*_*ij*_ and *G*_*ij*_ reflect the susceptance and conductance between the node *i* and *j*. *δ*_*ij*_ = *δ*_*i*_ − *δ*_*j*_ denotes a change in voltage angle. *N* represents the number of buses. More details of power flow formulas are discussed in sit [55].

The inequality constraints, confines the physical devices to certain limits, to assure the security of the power network. Furthermore, active power outputs, reactive power outputs, Shunt *VAR* compensators, transformer turn ratios, the voltage of all the generator units as well as slack should be limited by their upper and lower limits as formulated sit [39] [41]:

$$P_{Gi}^{mn}\leq P_{Gi}\leq P_{Gi}^{mx}i=1,2,3,\ldots \ldots \ldots N_{G}-1$$ (15)

$$T_{i}^{mn}\leq T_{i}\leq T_{i}^{mx}i=1,2,3,\ldots \ldots \ldots N_{T}$$ (16)

$$V_{Gi}^{mn}\leq V_{Gi}\leq V_{Gi}^{mx}i=1,2,3,\ldots \ldots \ldots N_{G}$$ (17)

$$Q_{Gi}^{mn}\leq Q_{Gi}\leq Q_{Gi}^{mx}i=1,2,3,\ldots \ldots \ldots N_{G}$$ (18)

$$Q_{ci}^{mn}\leq Q_{ci}\leq Q_{ci}^{mx}i=1,2,3,\ldots \ldots \ldots N_{c}$$ (19)

Security constraints, such as the voltage values of *PQ* buses and voltage of transmission line should be limited within the boundaries of its capacity. Which can be formulated as follows sit [36]:

$$V_{li}^{mn}\leq V_{li}\leq V_{li}^{mx}i=1,2,3,\ldots \ldots \ldots N_{L}$$ (20)

$$S_{linei}\leq S_{linei}^{mx}i=1,2,3,\ldots \ldots \ldots N_{l}$$ (21)

Similarly, the inequality constraints of the control variables, like voltage magnitude of *PV* bus, real and reactive power output at swing bus and generation, and loading of the transmission line can be combined into one objective part in the form of quadratic penalty expressions. Furthermore, a particular penalty factor is multiplied with the square of the control variable and then is added to the objective function sit [36]. Mathematical formula of the penalty function is stated as follows:

$$\begin{array}{c} J_{Avg}=J+\alpha_{p}{\left(P_{G_{i}}-P_{G_{i}}^{lim}\right)}^{2}+\alpha_{v}\sum_{i=1}^{NL}{\left(V_{L_{i}}-V_{L_{i}}^{lim}\right)}^{2}+\alpha_{q}\sum_{i=1}^{NG}+\alpha_{s}\sum_{i=1}^{nl}{\left(S_{l_{i}}-S_{l_{i}}^{max}\right)}^{2} \end{array}$$ (22)

Where *α*_*p*_,*α*_*v*_, *α*_*q*_ and *α*_*s*_ represent the penalty factors, *x*_*lim*_ is the boundary of the control variable. If *x* value crosses the upper limit, then it automatically brings x to the *x*_*lim*_, similarly, if *x* crosses the lower limit, then it brings to the *x*_*lim*_ sit [36]. Limits of the control variable can be expressed mathematically as follows:

$$\begin{array}{c} x_{lim}=\{\begin{array}{c} x^{max};x>x^{max}\\ x^{min};x<x^{min} \end{array} \end{array}$$ (23)

## 3 Particle swarm optimization (PSO) algorithm

The particle swarm optimization is a meta-heuristic population-based algorithm originally designed by Kennedy and Eberhart sit [56]. The technique is based on the combined behavior of living organisms such as a swarm of fish or a flock of birds. The PSO algorithm consists of two expressions *P*_*best*_ and *G*_*best*_ Its position (*X*) and velocity (*V*) updates in every iteration. These parameters can be expressed mathematically as follows:

$$\begin{array}{c} V_{i}\left(t+1\right)=wV_{i}\left(t\right)+c_{1}r_{1}(P_{best_{i}}\left(t\right)-X_{i}\left(t\right)+c_{2}r_{2}(g_{best_{i}}\left(t\right)-X_{i}\left(t\right) \end{array}$$ (24)

$$\begin{array}{c} X_{i}\left(t+1\right)=X_{i}+V_{i}\left(t+1\right) \end{array}$$ (25)

Where *w*, *c*_1_, and *c*_2_ are the inertia weight and acceleration coefficients, *r*_1_ and *r*_2_ denotes two random values within the range of [1, 0]. Inertia weight is calculated on linearly decreasing order based on number of iterations. Inertia weight can be calculated mathematically as follows sit [57] [58]:

$$\begin{array}{c} w=w^{max}-\frac{(w^{max}-w^{min})*iteration}{Max-iteration} \end{array}$$ (26)

The PSO algorithm can be studied in detail at sit [59].

## 4 Firefly optimization algorithm (FOA)

The firefly optimization algorithm is based on fireflies. These fireflies emit unique flashing light for their survival sit [49] [60]. The algorithm based on the intensity of flashing light and medium’s absorption. As stated by the inverse square law, the light strength decreases from a light source as distance increases. Moreover, the medium between light source and destination also absorbs the light. The method can be studied in more detail with the mathematical formulation in sit [61]

## 5 Hybrid firefly and particle swarm optimization (HFPSO) technique

**Fig 1:**
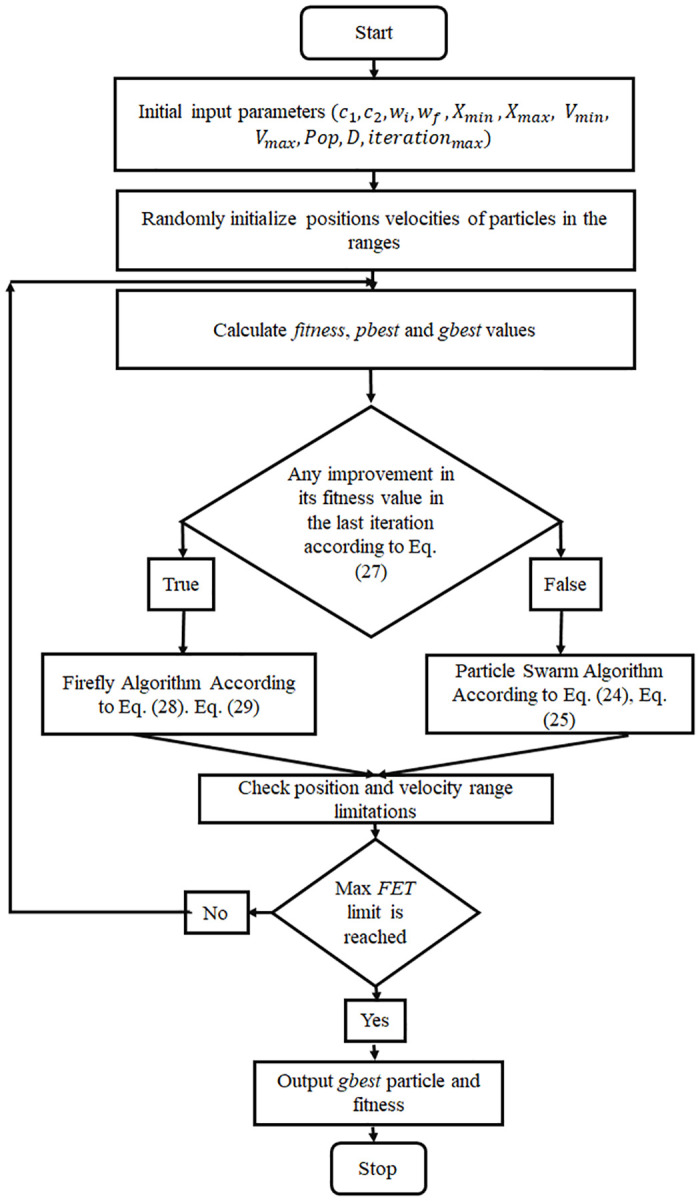
Flowchart demonstrates the optimization procedure of the basic HFPSO method sit [51].

The hybrid firefly and particle swarm optimization has been designed by Ibrahim Berkan Aydilek sit [51]. Hybrid equilibrium is maintained between exploration (*localoptima*) and exploitation (*globaloptima*) to take the strengths and advantages of both firefly and particle swarm methods sit [62] [63]. There are no velocity (*V*) and personal best location (*p*_*best*_) terms in the firefly algorithm. In a global search, The PSO method offers fast convergence in terms of exploration. Moreover, the firefly algorithm is beneficial in local region search or it gives fine exploitation. The flowchart of the HFPSO method is shown in Fig 1.

Initially, input parameters are inserted. Then these parameters are used step by step by both population-based methods. Afterward, constant swarm vectors are initiated in the search space and velocity ranges. Global best (*g*_*best*_) and individual best (*p*_*best*_) swarms are mathematically considered and allocated. The calculated values are compared in the final alternation. Moreover, the present location is saved and then new velocity and location are calculated as follows sit [51]:

$$\begin{array}{c} f\left(i,t\right)=\{\begin{array}{cc} true,iffitness-value(particle_{i}^{t}\leq g^{best^{t-1}}) & \\ false,iffitness-value(particle_{i}^{t}\leq g^{best^{t-1}}) & \end{array} \end{array}$$ (27)

$$\begin{array}{c} X_{i}\left(t+1\right)=X_{i}B_{o}e^{rr^{2}ij}-\left(X\right)i-g^{best^{t-1}})+a\epsilon \end{array}$$ (28)

$$\begin{array}{c} V_{i}\left(t+1\right)=X_{i}\left(t+1\right)-X_{i_{temp}} \end{array}$$ (29)

If a particle’s fitness value is equal to or better than the preceding global best, then the particle will be picked up by the firefly part according to the Eqs (28) and (29); otherwise, it will be carried by the PSO part according to the Eqs (24) and (25).

## 6 Application of the HFPSO method to optimal load flow problems

The subsequent steps show the application procedure of the proposed HFPSO algorithm to deal with the optimal power flow problem.

**Step 1** Define the system data, real power limits, reactive power limits, generators’ data, state the primary values of real power and the voltage level of generator buses, reactive power of shunt capacitors, and the turn ratio of transformers.

**Step 2** Execute the base case power flow. Evaluate the initial values of the objective functions that include the generation cost, voltage profile improvement, voltage stability enhancement, and real and reactive power transmission line loss reduction, by applying Eqs (1), (3), (4), (6), and (7).

**Step 3** State the *i* − *th* goal function *f*_*i*_ to evaluate as described in section 2. Define the designed variables (*X*) and its limits (*X*_*min*_, *X*_*max*_), initial population (*Pop*), dimensions (*D*), maximum iterations (*Iteration*_*max*_), and algorithm specified parameters(*C*, *w* and *V*).

**Step 4** Generate prime random positions of swarm particles (*population*) within specified limits of controlled variables. The position of the particles are formulated in such a way:

$$\begin{array}{c} Swerm-population=(\begin{array}{ccc} X_{11} & \ldots & X_{1n}\\ \vdots & \ddots & \vdots \\ X_{m1} & \cdots & X_{mn} \end{array}) \end{array}$$ (30)

*k* = 1, 2, 3… *m* and *j* = 1, 2, 3… *n*.

Where the control variables and the number of various solutions are denoted by *n* and *m*. The estimation of the *j* − *th* designed variable X_(*k*,*j*)_ and *k* − *th* applicant solution can be calculated as follows:

$$\begin{array}{c} X_{k,j}=X_{j}^{\text{min}}+rand\left(.\right)[X_{j}^{\text{max}}-X_{j}^{\text{min}}] \end{array}$$ (31)

Where $X_{j}^{\left(max\right)}$ and $X_{j}^{\left(min\right)}$ are the limits of the *j* − *th* designed variables and *rand*(.) denotes the random number within limits of (0 − 1). For more clarification, the physical components of *X*_(*k*, *j*)_ can be formulated as follow:

$$Swerm-population=(\begin{array}{c} P_{G\text{1,}2},\ldots \ldots P_{G\text{1,}N_{GG}},T_{1,1},\ldots \ldots T_{\text{1,}N_{TT}},V_{G\text{1,}1},\ldots \ldots V_{\text{1,}N_{GG}},Q_{C\text{1,}1},\ldots \ldots Q_{\text{1,}N_{CC}}\\ P_{G\text{2,}2},\ldots \ldots P_{G\text{2,}N_{GG}},T_{2,1},\ldots \ldots T_{\text{2,}N_{TT}},V_{G\text{2,}1},\ldots \ldots V_{\text{2,}N_{GG}},Q_{C\text{2,}1},\ldots \ldots Q_{\text{2,}N_{CC}}\\ \vdots \\ P_{Gm,2},\ldots \ldots P_{Gm,N_{GG}},T_{m,1},\ldots \ldots T_{m,N_{TT}},V_{Gm,1},\ldots \ldots V_{m,N_{GG}},Q_{Cm,1},\ldots \ldots Q_{m,N_{CC}} \end{array})$$ (32)

**Step 5** Execute the load flow for every single solution and compute the value of the particular objective function that relates to the solution.

**Step 6** Evaluate the fitness value and find the personal best (*pbest*) and global best (*gbest*) solutions in the group of calculated values.

**Step 7** Examine the improvement in the calculated objective function values in the final iteration as stated by Eq (27).

**Step 8** Calculate the dispatch in view of the changed vector of controlled variables. Compute the fresh values of the objective functions. Include the allocated penalty(s) to the goal function, if it violates the limits, according to Eq (22).

**Step 9** Compare the goal function *f*_*i*_ values. If the values are superior to previous ones, then execute the Eqs (28) and (29); otherwise, use the Eqs (24) and (25), respectively.

**Step 10** If the termination standard is achieved, then stop and print the results of the optimal values. Otherwise, come back to step 7.

**Fig 2:**
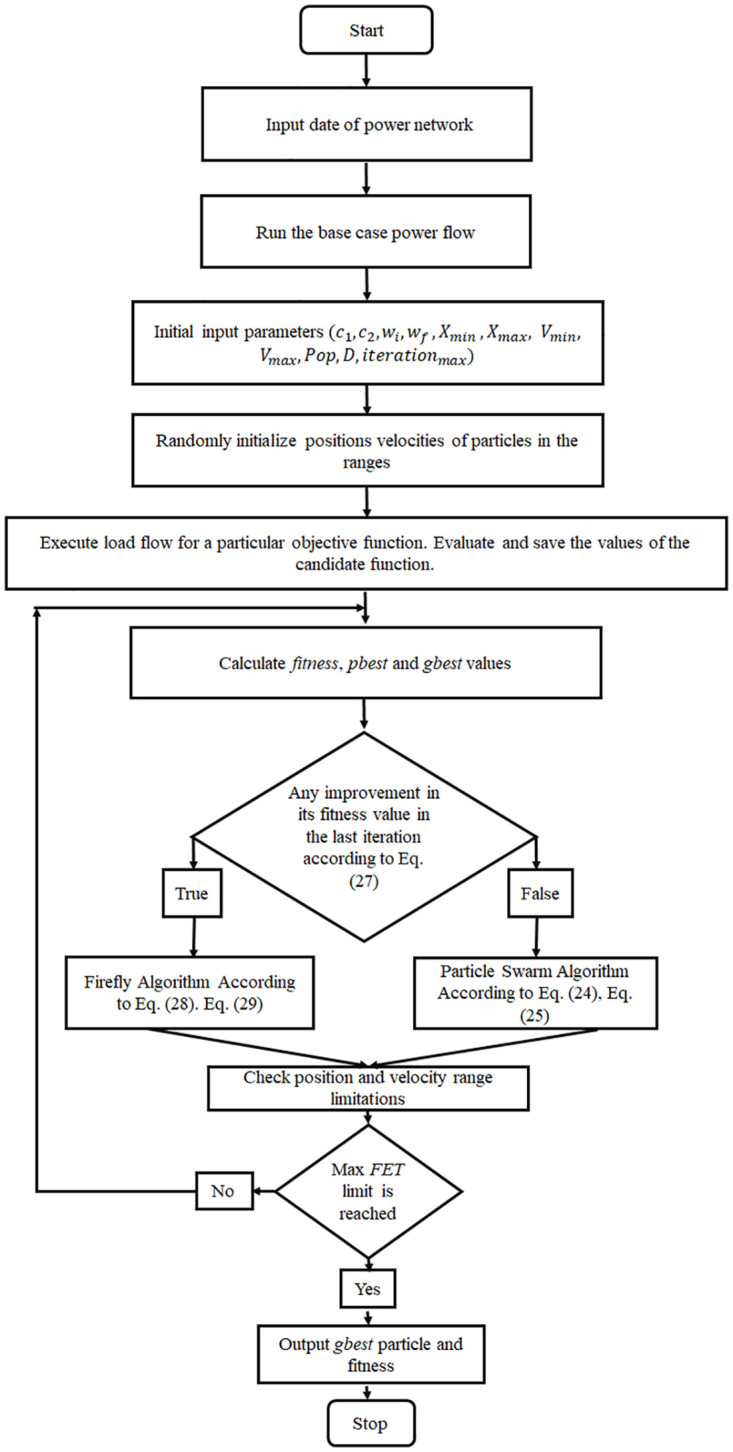
The flowchart demonstrates the application of the HFPSO method for OPF problems.

For more clarification, the flowchart of the suggested application of the HFPSO method to solve optimal load flow is presented in Fig 2.

## 7 Results and discussion

Standard *IEEE* − 30 bus test network is used as a benchmark function for single-objective OPF problems to examine the efficiency of the proposed HFPSO and original PSO algorithms. Both algorithms were initialized with a population of 30 and executed for a maximum iteration of 100. The algorithms are coded and executed in *MATLAB* *R*2016*asitbib* [64] *andtheresultsarecarriedoutonaPCwith8GBRAManda4GHzIntelCorei7CPU*.

### 7.1 IEEE 30-Bus test network

**Fig 3:**
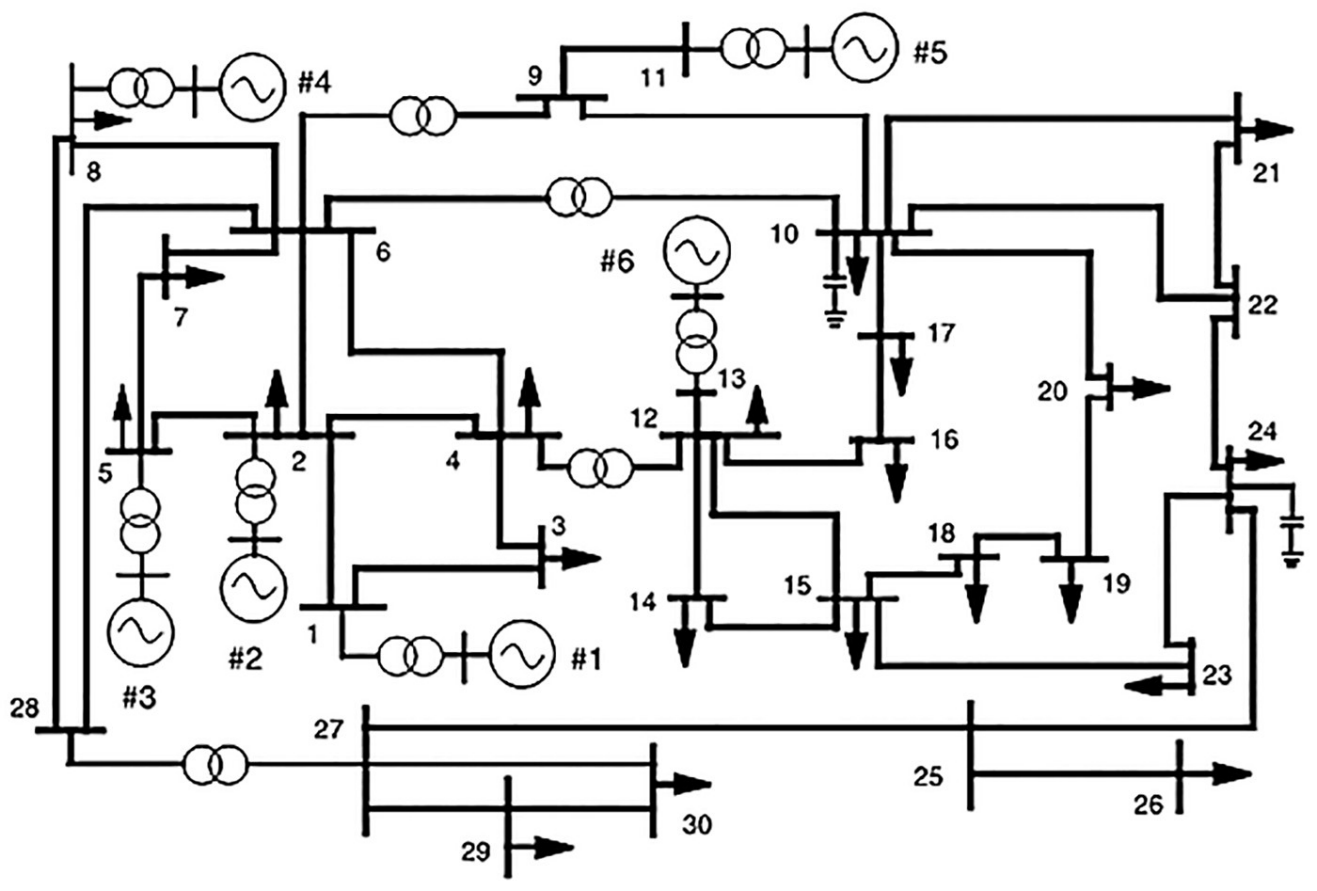
One-line diagram of standard IEEE 30-bus test network.

In this research work, the *IEEE*30 − *bus* test scheme is applied for the suggested HFPSO and the original PSO algorithms to investigate the effectiveness of the suggested HFPSO method. Fig 3 shows one-line diagram of the IEEE 30-bus test system with the following characteristics sit [36] [5]: The system has 6 generator units at buses 1, 2, 5, 8, 11, and 13 of the network. Also, four tap-controlled transformers are connected between the transmission lines 6 to 9, 6 to 10, 4 to 12, and 27 28, in voltage limits of (0.9 − 1.1). Reactive power sources in *MVAR*(0 − 5) are installed at the 10, 12, 15, 17, 20, 21, 23, 24, and 29 load buses. Moreover, the voltage magnitudes of *PV* buses are limited from 0.95 to 1.1(*p*.*u*.). Operating limits of the load buses are subjected from 0.95 to 1.05(*p*.*u*.). In addition, the bus data, line data, and generator cost coefficients are detailed in sit [5].

To validate the usefulness and robustness of the proposed method, several cases with diverse goal functions, such as total fuel cost reduction, voltage deviation, voltage profile enhancement *L*_*max*_, real power losses, and reactive power losses have been simulated as follows:

#### 7.1.1 Case 1: Fuel cost minimization

**Fig 4:**
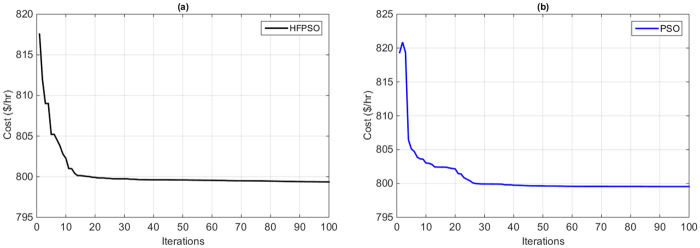
Convergence curves of total fuel cost minimization based on (a) the HFPSO algorithm and (b) the PSO algorithm.

In this section, the minimization of the total fuel cost of generation is considered as a goal function during the execution of the HFPSO and the original PSO method. As we see, from the graphs (a) and (b) in Fig 4, the proposed algorithm requires only 25 iterations while the original PSO method needs 40 iterations to reach the optimal solution. The proposed algorithm also achieves a fine convergence rate as compared to the original PSO method. Optimum solutions and values of the control variables of the methods are shown in Tables 1 and 2. In addition, the fuel cost value calculated by the proposed method 11.4% decreased from the base value 902.0207 $/*h* sit [36] to the optimized value of 799.123 $/*h* with an average execution time of a single repetition of 0.821s. Table 3 illustrates the improved performance of the HFPSO method over the current heuristic optimization methods in terms of an optimum solution. The minimum values achieved by the proposed algorithm are 799.132, as compared to the best value achieved by the MVO algorithm is 799.242. Consequently, these results showed the dominance of the HFPSO heuristic algorithm over the current heuristic methods in terms of optimality and convergence.

**Table 1:** Optimum tuning of the dependent variables for various cases using the HFPSO technique (standard IEEE 30-bus test network).

Control Variable	Limits		Initial Status	HFPSO Algorithm				
	Min	Max		Case 1	Case 2	Case 3	Case 4	Case 5
P_*G*1_(*MW*)	50	200	99.248	176.838	176.3094	173.1729	51.2668	51.3085
P_*G*2_(*MW*)	20	80	80	49.1003	49.5793	48.0205	80	80
P_*G*5_(*MW*)	15	50	50	21.2822	20.715	19.7516	50	35
P_*G*8_(*MW*)	10	35	20	20.9561	21.8718	20.5156	35	50
P_*G*11_(*MW*)	10	30	20	11.8619	12.7804	18.4118	30	35
P_*G*13_(*MW*)	12	40	20	12	12	12.0041	40	40
V_*G*1_(*p*.*u*)	0.95	1.1	1.05	1.1	1.0434	1.1	1.1	1.1
V_*G*2_(*p*.*u*)	0.95	1.1	1.04	1.0876	1.0259	1.089	1.1	1.1
V_*G*5_(*p*.*u*)	0.95	1.1	1.01	1.0585	1.0106	1.0474	1.082	1.0919
V_*G*8_(*p*.*u*)	0.95	1.1	1.01	1.0708	1.008	1.0702	1.089	1.1
VG_11_(*p*.*u*)	0.95	1.1	1.05	1.1	1.0128	1.0682	1.1	1.1
V_*G*13_(*p*.*u*)	0.95	1.1	1.05	1.1	0.9944	1.1	1.1	1.1
T_6,9_	0.9	1.1	1.078	1.0344	1.0217	0.9848	1.0538	1.0018
T_6,10_	0.9	1.1	1.069	0.9216	0.9	0.9	0.9	0.9657
T_4,12_	0.9	1.1	1.032	0.9994	0.957	0.967	0.9809	0.9949
T_28,27_	0.9	1.1	1.068	0.9726	0.9685	0.9578	0.9727	0.9863
Q_*C*10_(Mvar)	0	5	0	3.457	4.4104	4.263	5	5
Q_*C*12_(*Mvar*)	0	5	0	2.8844	3.8688	4.9858	5	5
Q_*C*15_(*Mvar*)	0	5	0	3.8167	5	5	5	5
Q_*C*17_(*Mvar*)	0	5	0	4.7886	2.0503	5	5	5
_Q*C*20_(*Mvar*)	0	5	0	4.8039	2.071	5	5	5
_Q*C*21_(*Mvar*)	0	5	0	5	4.0697	2.9266	5	5
_Q*C*23_(*Mvar*)	0	5	0	3.6471	5	5	5	5
_Q*C*24_(*Mvar*)	0	5	0	5	5	4.351	5	5
_Q*C*29_(*Mvar*)	0	5	0	2.6582	2.4285	4.8809	2.6549	3.3162
**Cost(($/h)**	-	-	902.0207	**799.123**	803.6002	800.8403	999.81	967.2057
**PLoss (*MW*)**	-	-	5.8482	8.6375	9.8538	8.4762	**2.8652**	2.9101
**QLoss(*Mvar*)**	-	-		-3.1221	5.3669	-3.1991	-24.491	**-25.204**
**TVD**	-	-		1.7216	**0.1163**	1.9787	2.04	2.1318
**Lmax**	-	-		0.1186	0.137	**0.144**	0.1156	0.1142

The boldface values describe optimized values

**Table 2:** Optimum solutions and tuning of the dependent variables for various cases based on the PSO technique (standard IEEE 30-bus test network).

Control Variable	Limits		Initial Status	PSO Algorithm				
	Min	Max		Case 1	Case 2	Case 3	Case 4	Case 5
P_*G*1_(*MW*)	50	200	99.248	176.385	168.9926	164.4909	51.4181	52.0175
P_*G*2_(*MW*)	20	80	80	49.4459	48.2497	46.7857	80.0000	79.8978
P_*G*5_(*MW*)	15	50	50	21.8773	20.2761	23.3709	49.9872	49.9998
P_*G*8_(*MW*)	10	35	20	21.6395	24.1536	16.5316	30.0000	29.8163
P_*G*11_(*MW*)	10	30	20	11.2976	18.9398	13.0738	30.0000	29.8163
P_*G*13_(*MW*)	12	40	20	12.2698	12.0265	27.4438	39.9944	40.0000
V_*G*1_(*p*.*u*)	0.95	1.1	1.05	1.0541	1.0380	1.0999	1.0999	1.1000
V_*G*2_(*p*.*u*)	0.95	1.1	1.04	1.0342	1.0276	1.0942	1.0981	1.1000
V_*G*5_(*p*.*u*)	0.95	1.1	1.01	1.0014	1.0037	1.0478	1.0815	1.0858
V_*G*8_(*p*.*u*)	0.95	1.1	1.01	1.0057	1.0108	1.0625	1.0899	1.1000
V_*G*11_(*p*.*u*)	0.95	1.1	1.05	1.0291	0.9984	1.0904	1.0987	1.0376
V_*G*13_(*p*.*u*)	0.95	1.1	1.05	1.0484	1.0162	1.0819	1.1000	1.0688
T_6,9_	0.9	1.1	1.078	0.9429	0.9624	0.9757	1.1000	1.0603
T_6,10_	0.9	1.1	1.069	1.0539	0.9047	0.9402	0.9001	1.0391
T_4,12_	0.9	1.1	1.032	0.9959	0.9776	0.9071	1.0331	1.0241
T_28,27_	0.9	1.1	1.068	0.9692	0.9716	0.9414	1.0089	1.0363
Q_*C*10_(*Mvar*)	0	5	0	2.0825	3.5950	0.0926	4.9997	0.3746
Q_*C*12_(*Mvar*)	0	5	0	1.7209	0.3615	1.0002	0.0122	4.9986
Q_*C*15_(*Mvar*)	0	5	0	4.0925	2.4505	3.7573	0.2437	4.9999
Q_*C*17_(*Mvar*)	0	5	0	1.2855	1.3195	1.0310	0.0375	1.3503
Q_*C*20_(*Mvar*)	0	5	0	3.2046	2.9503	3.1403	4.9997	4.9548
Q_*C*21_(*Mvar*)	0	5	0	4.1781	0.0876	3.2247	0.1737	0.6480
Q_*C*23_(*Mvar*)	0	5	0	1.7577	3.2800	4.2165	4.9961	2.7229
Q_*C*24_(*Mvar*)	0	5	0	1.6139	3.5760	0.5942	0.2291	4.9995
Q_*C*29_(*Mvar*)	0	5	0	3.9931	3.5271	4.7442	4.9967	1.4439
**Cost(($/h)**	-	-	902.0207	**799.5433**	805.0754	807.8701	967.287	966.95
**PLoss(MW)**	-	-	5.8482	8.7158	9.3298	9.5863	**2.9473**	2.9101
**QLoss(Mvar)**	-	-		5.3430	9.9265	9.2386	-21.329	**-23.756**
**TVD**	-	-		1.2340	**0.1467**	1.3401	1.8220	0.9126
**Lmax**	-	-		0.1256	0.1389	**0.1170**	0.1179	0.1323

The boldface values describe optimized values

**Table 3:** Assessment of the solutions achieved for total fuel cost reduction (Standard IEEE 30-bus test system).

Method	Fuel Cost ($/h)	Algorithm Description
MVO sit [65]	799.242	Multi-verse Optimizer
Jaya sit [38]	800.479	Jaya Algorithm
PSO	**799.543**	Particle Swarm Optimization
DE sit [41]	799.289	Differential Evolution
BHBO sit [36]	799.921	Black Hole Based Optimization
HFPSO	**799.123**	Hybrid Firefly Particle Swarm Optimization

#### 7.1.2 Case 2: Voltage profile improvement

**Fig 5:**
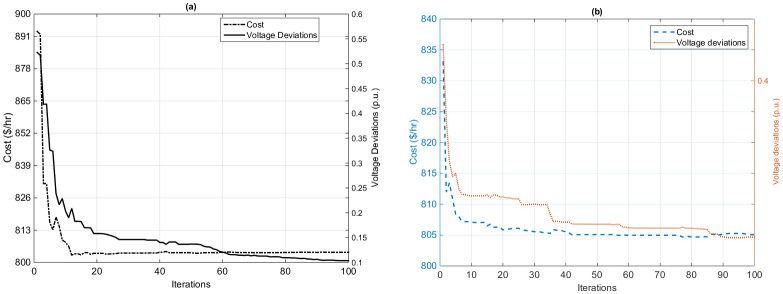
Convergence curves of the voltage profile improvement by using (a) the HFPSO algorithm and (b) the PSO algorithm.

The objective of this section is to minimize the total fuel cost ($/*h*) of the system and to improve the voltage profile simultaneously by limiting the voltage deviation (*p*.*u*.) of the load buses (*PQ* buses) from the reference of 1.0*p*.*u*. during the execution of the proposed HFPSO and original PSO algorithms. Fig 5(*a*) and 5(*b*) describe the convergence curves of fuel cost ($/*h*) and voltage deviation (*p*.*u*.) minimization based on current methods. The graphical representation shows that the proposed algorithm achieves a good convergence rate. Also, the proposed method needs only 20 and 91 iterations, for the fuel cost and voltage deviation, while the original PSO method requires 100 iterations to achieve the optimal solution. The optimum solutions and control variables for the case obtained by the proposed and the original PSO algorithms are tabulated in Tables 1 and 2. Table 1 shows that the voltage deviation is significantly minimized as related to the base value sit [36]. The deviation 89.85% decreased from the base value 1.1469 p.u. to an optimum value of 0.1163 p.u. based on the proposed technique, while the deviation decreased only 59.28% from the base value of 1.1469 (*p*.*u*) to the global value of 0.467 (*p*.*u*) based on the original PSO method.

To further verify the effectiveness of the suggested algorithm, the optimum solution of the algorithm is also compared with the various natural-inspired-heuristic algorithms in the present research work, as shown in Table 4. Consequently, optimum solutions to the fuel cost and voltage deviation obtained from the proposed HFPSO technique are better than the original PSO and most of the heuristic methods.

**Table 4:** Examination of the solutions gained for voltage profile improvement (Standard IEEE 30-bus test system).

Algorithm	Voltage Deviation	Algorithm Description
MVO sit [65]	0.1056	Multi-verse Optimizer
FA	0.1474	Firefly Algorithm
PSO	**0.1467**	Particle Swarm Optimization
DE sit [41]	0.1357	Differential Evolution
BHBO sit [36]	0.1262	Black Hole Based Optimization
HFPSO	**0.1163**	Hybrid Firefly Particle Swarm Optimization

#### 7.1.3 Case 3: Voltage stability enhancement

**Fig 6:**
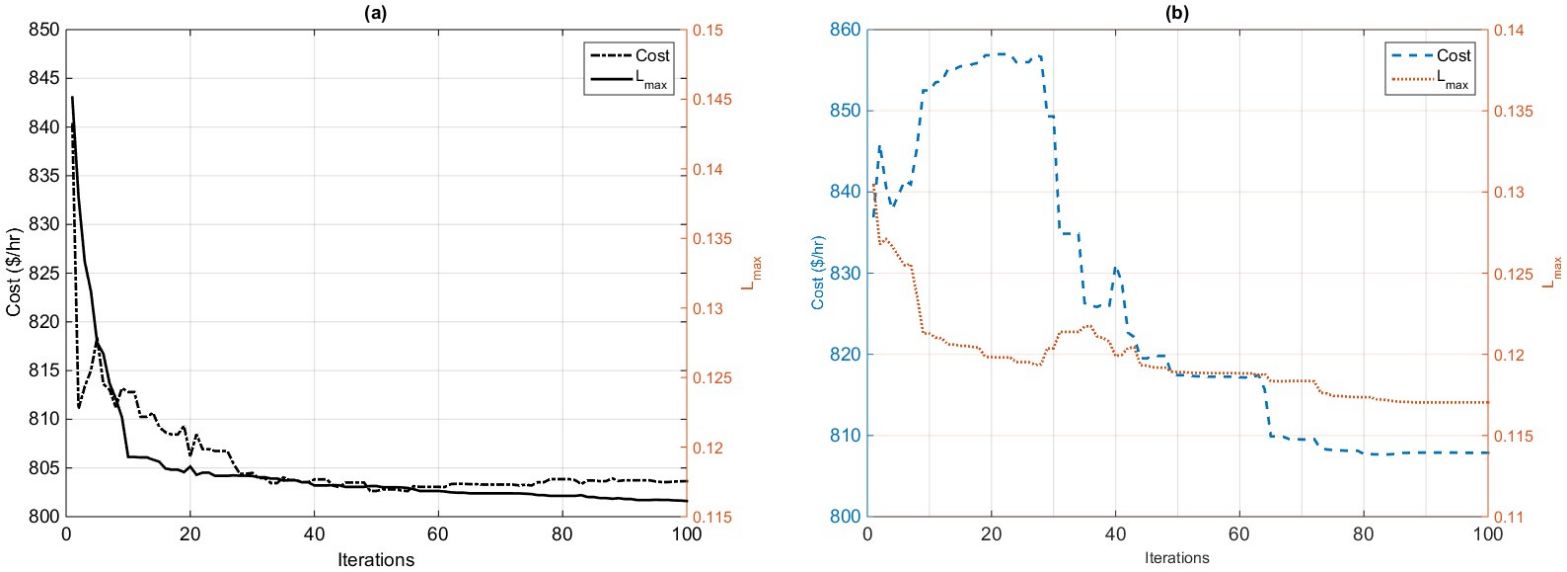
Convergence curves of voltage stability enhancement based on (a) the HFPSO algorithm and (b) the PSO algorithm.

In this section, fuel cost and voltage stability enhancement are chosen as a single-objective function to be improved based on the proposed HFPSO and the original PSO algorithms as shown in Fig 6. The proposed algorithm achieves a very good convergence rate again, as compared to the original PSO method as illustrated in Fig 6(*a*) and 6(*b*).

It is important to note that the voltage stability index is strengthened by 33.94% from the base value of 0.1723 sit [36] to the optimum value of 0.1144, by the proposed algorithm.

It is evident from Fig 6(*b*) that the original PSO algorithm acquired an abrupt and very weak convergence ratio. Furthermore, the stability index is reinforced by 32.09% from the base value 0.1723 to the improved value 0.1170. Table 5 compares the results of the previous population-based methods with the optimal value achieved by the application of the proposed HFPSO technique. It is obvious from Table 5 that the minimum value obtained by the proposed algorithm is 0.1144, as compared to the best minimum value obtained by the MVO algorithm 0.1146 from the current literature work. So, it is clear from the results and comparisons that the proposed algorithm is very efficient to solve the OPF problems.

**Table 5:** Evaluation of the solutions gained for voltage stability enhancement (Standard IEEE 30-bus test system).

Algorithm	Lmex	Algorithm Description
MVO sit [65]	0.1146	Multi-verse Optimizer
Jaya sit [38]	0.1243	Jaya Algorithm
PSO	**0.1170**	Particle Swarm Optimization
DE sit [41]	0.1219	Differential Evolution
BHBO sit [36]	0.1167	Black Hole Based Optimization
HFPSO	**0.1144**	Hybrid Firefly Particle Swarm Optimization

#### 7.1.4 Case 4: Active power transmission Losses reduction

**Fig 7:**
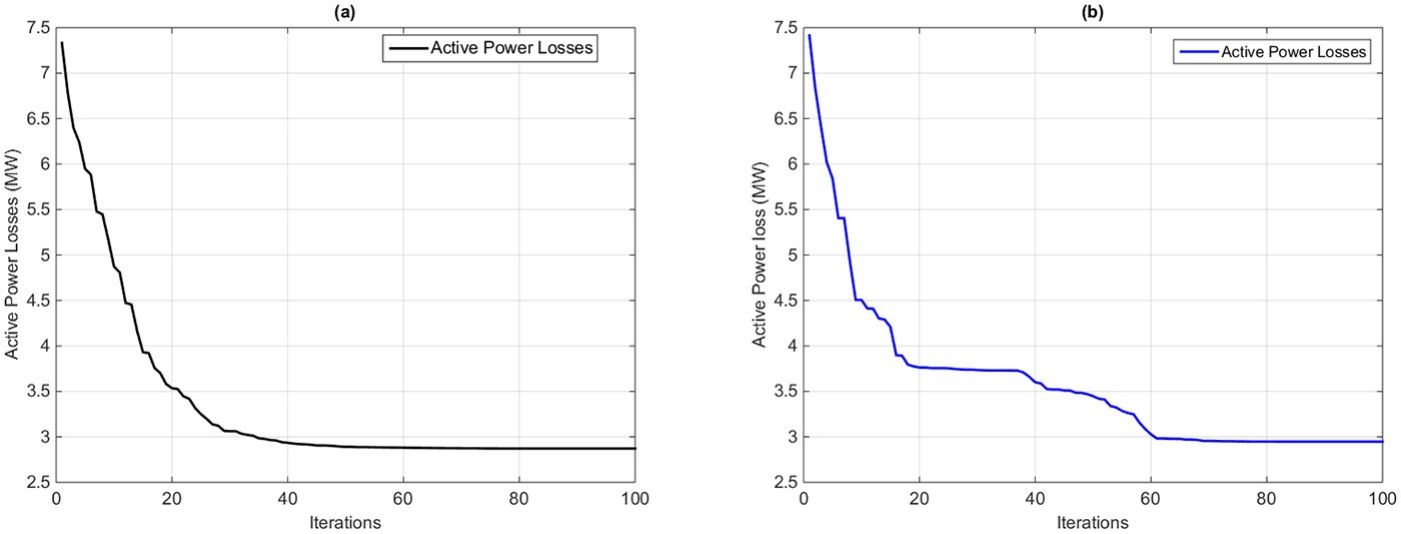
Convergence curve of real power transmission line loss minimization by using (a) the HFPSO algorithm and (b) the PSO algorithm.

This section explains the active power loss optimization as a single-objective function by using the proposed HFPSO and original PSO algorithms. Fig 7 illustrates the sketched graphs of the objective function over repetitions. Fig 7(*a*) shows that the proposed algorithm achieved an optimal solution in 40 iterations and has fast convergence compared to the 60 iterations of the PSO. Table 1 arranges optimum solutions and control variables achieved by using the HFPSO algorithm. The active power transmission line losses are reduced 50.78% from the base value of 5.821 MW sit [36] to the optimal value of 2.865MW. Optimum solutions and control variables of the PSO algorithm are tabulated in Table 2. Real power losses are minimized by only 49.37% from the base case 5.821 MW to the best value of 2.947 MW. The real power losses from the previous heuristic techniques in Table 6 are also matched with the proposed HFPSO method to demonstrate its effectiveness. The minimum value of the proposed algorithm is 2.865, as compared to the global minimum value by MVO algorithm 2.881 from the current literature work.

**Table 6:** Comparison of the results obtained for active power losses reduction (Standard IEEE 30-bus test system).

Algorithm	Real Power Losses (MW)	Algorithm Description
MVO sit [65]	2.881	Multi-verse Optimizer
Jaya sit [38]	3.101	Jaya Algorithm
PSO	**2.947**	Particle Swarm Optimization
BHBO sit [36]	3.503	Black Hole Based Optimization
HFPSO	**2.865**	Hybrid Firefly Particle Swarm Optimization

#### 7.1.5 Case 5: Minimization of reactive power transmission losses

**Fig 8:**
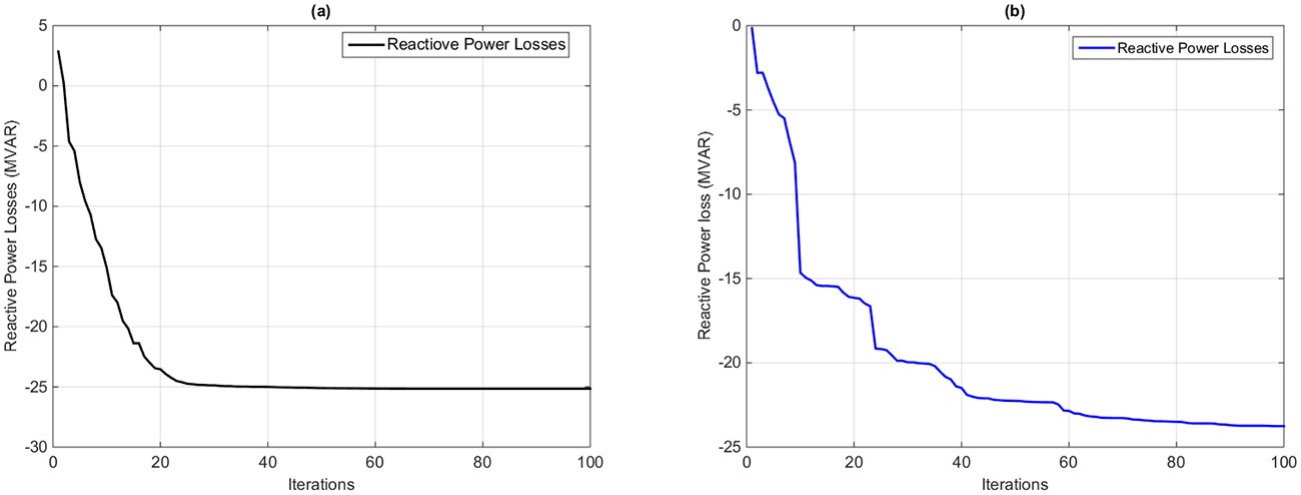
Convergence curve of reactive power transmission line loss minimization based on (a) the HFPSO algorithm and (b) the PSO algorithm.

The main goal of this section is to reduce the reactive power losses of the transmission lines based on the proposed HFPSO technique and compare the optimum solution with the original PSO algorithm. Fig 8 shows the convergence curves of the reactive power losses as an objective function in this case. It is observed from Fig 8(*a*) and 8(*b*) that the proposed algorithm achieved an optimum solution in only 22 iterations with a fine convergence ratio as compared to the original PSO method. The control variables and optimal solutions obtained by using the HFPSO and PSO algorithms are mentioned in Tables 1 and 2. The reactive power losses are minimized from the base case value of -4.6066 MVAR sit [36] to the optimal value of -25.204 MVAR by using the HFPSO technique. But the same losses are only reduced to -21.329 MVAR after applying the PSO method. Table 7 compares the optimal values of the same losses of the population-based techniques from the current research work with the proposed method to further validate the usefulness of the proposed algorithm. As we see, the value of the MVO algorithm is -25.038 and is only more optimized as compared to the optimum value of the proposed algorithm.

**Table 7:** Comparison of the solutions obtained for reactive power losses minimization (Standard IEEE 30-bus test system).

Algorithm	Reactive Power Losses (MWAR)	Algorithm Description
MVO sit [65]	-25.038	Multi-verse Optimizer
FA	-20.464	Firefly Algorithm
PSO	**-21.329**	Particle Swarm Optimization
BHBO sit [36]	-20.152	Black Hole Based Optimization
HFPSO	**-25.204**	Hybrid Firefly Particle Swarm Optimization

### 7.2 Statistical results and complexity

To check the robustness of the algorithm, 40 independent trials are performed with initial populations and iterations of 50 and 100. Table 8 shows the best, average, worst, and standard deviation values. It can be observed from the table that best, the mean, and the worst values are very close to each other and the standard deviation value is the minimum, which concludes the robustness of the HFPSO algorithm.

**Table 8:** Statistcal calculations over 40 independent triels of HFPSO algorithm.

Cases	Best	Average	Worse	Standard Deviation	Average CPU Time (s)
**Case 1**	799.1133	**799.1232**	799.535	0.0064	24.2
**Case 2**	0.11433	**0.11636**	0.11848	0.0075	25.3
**Case 3**	0.11345	**0.11443**	0.11539	0.0468	43.8
**Case 4**	2.8344	**0.8657**	2.8834	0.0084	43.8
**Case 5**	-25.2235	**-25.2042**	-25.1863	0.00186	42.4

We are interested in computing the computational complexity of the algorithm. More precisely, we compute the temporal (or time) complexity which indicates how the computational time of the algorithm changes with a change in input parameters.

FA and PSO techniques have two inside loops, when passing over the population of size *n* and one outside loop for *t* cycles. Both techniques have time complexity of *O*(*n*^2^ *t*) in the extreme case. When *n* is relatively large, we can rank the selecting parameters for all particles by applying sorting technique to decrease the complexity to *O*(*ntlog*(*n*)) sit [66] [67].

These two algorithm have the same order of complexity and are applied in HFPSO simultaneously. The overall complexity in the extreme case (resp. when *n* is relatively large) of the algorithm is therefore *O*(*MaxFESn*^2^ *t*) (resp. *O*(*MaxFESntlog*(*n*))) since the algorithm runs until the maximum number of function evaluations (MaxFES) is reached.

## 8 Conclusions

In this article, a novel meta-historic optimization algorithm called HFPSO has been effectively applied to handle the OPF issues in power systems. Equilibrium is maintained between explorations and exploitation to take the advantages of both FOA and PSO methods. Various objective functions of OPF problems were considered: total fuel cost reduction, voltage stability enhancement, voltage profile improvement, active power transmission line loss minimization, and reactive power transmission line loss minimization. A standard IEEE 30-bus test network was tested to authenticate the validity of the HFPSO to solve the OPF problems. The results of the HFPSO algorithm were compared with the standard PSO algorithm and other optimization techniques. Results revealed that optimal solution for each considered case could be presented by the HFPSO algorithm. The new suggested idea of the HFPSO technique led to fast finding of a global solution (that is, supported the exploration and exploitation property). Furthermore, results showed the effectiveness of HFPSO technique concerning the satisfactory convergence rate. Statistical analysis showed that HFPSO algorithm is a robust and reliable optimization method to solve OPF problems. In conclusion, based on the applicability, and performance of the HFPSO, it can be said that this method offers an excellent tool to solve OPF issues of power networks.
